# First Evidence for the Presence of Iron Oxidizing Zetaproteobacteria at the Levantine Continental Margins

**DOI:** 10.1371/journal.pone.0091456

**Published:** 2014-03-10

**Authors:** Maxim Rubin-Blum, Gilad Antler, Rami Tsadok, Eli Shemesh, James A. Austin, Dwight F. Coleman, Beverly N. Goodman-Tchernov, Zvi Ben-Avraham, Dan Tchernov

**Affiliations:** 1 The Leon H. Charney School of Marine Sciences, University of Haifa, Haifa, Israel; 2 Department of Earth Sciences, University of Cambridge, Cambridge, United Kingdom; 3 Institute for Geophysics, Jackson School of Geosciences, University of Texas at Austin, Austin, Texas, United States of America; 4 Graduate School of Oceanography, The University of Rhode Island, Narragansett, Rhode Island, United States of America; 5 Department of Geophysical, Atmospheric and Planetary Sciences, Faculty of Exact Sciences, Tel Aviv University, Ramat Aviv, Israel; University of California, Merced, United States of America

## Abstract

During the 2010–2011 *E/V Nautilus* exploration of the Levantine basin’s sediments at the depth of 300–1300 m, densely patched orange-yellow flocculent mats were observed at various locations along the continental margin of Israel. Cores from the mat and the control locations were collected by remotely operated vehicle system (ROV) operated by the *E/V Nautilus* team. Microscopic observation and phylogenetic analysis of microbial 16S and 23S rRNA gene sequences indicated the presence of zetaproteobacterial stalk forming *Mariprofundus* spp. – like prokaryotes in the mats. Bacterial tag-encoded FLX amplicon pyrosequencing determined that zetaproteobacterial populations were a dominant fraction of microbial community in the biofilm. We show for the first time that zetaproteobacterial may thrive at the continental margins, regardless of crustal iron supply, indicating significant fluxes of ferrous iron to the sediment-water interface. In light of this discovery, we discuss the potential bioavailability of sediment-water interface iron for organisms in the overlying water column.

## Introduction

The recently described class of marine microbial iron oxidizers (FeOB), the zetaproteobacteria [1], is represented by a stalk-forming prokaryote *Mariprofundus ferrooxydans* [2], prevails in the marine iron-oxidizing biofilms [1], [3]–[7], and is mainly linked to hydrothermal activity [4], [5], [8] and exposed oceanic crust [9], [10]. Moreover, zetaproteobacteria were recently shown to participate in corrosion processes in the nearshore environments [3], [6] as well as exist in saline estuaries [11]. The autotrophic zetaproteobacteria may play a considerable role in global Fe and carbon cycling [4], although their biogeography and metabolic potential are still not fully understood. Since zetaproteobacteria studies are mostly limited to the deep sea, the iron-dependent carbon fixation is yet to be suggested as a considerable source of organic material in the benthic environment, and therefore the effects of FeOB on benthic-pelagic iron exchange are yet unexplored.

At sites of hydrothermal fluid emission and exposed ocean crust, high fluxes of ferrous iron from the crustal source provide metabolic energy to FeOB [4]. In the absence of the latter, diagenetic chemical reactions at the sediment-water interface and at the top sediment layers control fluxes of important metabolites, such as reduced iron [12], [13]. Recently, significant benthic iron fluxes were recognized in the iron isotopic values measured within *in situ* benthic chambers, which emphasized the importance of the microbial iron reduction within sediment for the iron supply to the sediment surface and the water column [12]. Given significant benthic iron fluxes and oxygenated conditions at the sediment-water interface, a niche for the FeOB can be formed at the continental margin, regardless of the crustal iron source.

The net outward flux of soluble iron from the sediment to the upper water column layers under upwelling or mixing conditions, measured at the continental margins, can provide supplementary nutrition to the primary producers and diazotrophs [12], [14]–[16]. These findings challenge the paradigm stating that the iron supply to the surface waters is controlled by the iron-rich dust supply [12], [17], [18]. In turn, the flux of soluble iron is controlled by the organic load that is causing rapid depletion of oxygen and switch to manganous and ferruginous respiration closer to the sediment-water interface, or by bioturbation/bioirrigation that allow for a rapid advection of the ferrous iron to the sediment-water interface [12], [13]. Moreover, physical or biological resuspension of the sediment also leads to the release of soluble particles to the medium overlaying the sediment [13], [19]. On the other hand, the microbially-enhanced iron oxide precipitation at the sediment-water interface can alter the bio-availability of the sediment-recycled iron, due to different solubility properties of various iron oxides [20]. FeOB precipitate nanoparticulate ferrihydrite - like phases with short-range structural order [21], although more organized iron minerals, such as lepidocrocite, were found in association with FeOB [22]. FeOB formed iron oxides are known to be excellent substrates for the iron-reducing bacteria [9], yet their bioavailability to other species is still unknown.

During the 2010–2011 exploration season of the *E/V Nautilus* we studied the deep benthic environment of the Levantine basin, the most oligotrophic part of the Mediterranean Sea [23]. The contrast between the high iron demand for required for nitrogen fixation [24] and the low iron availability in the surface waters [25] may explain the low nitrogen fixation rates in this area [26]. The paradigm of Fe-rich dust supply to surface waters as the dominant iron source includes Levantine basin [27]. In this study we show that zetaproteobacterial mats are present throughout Israel’s continental margins, at depths of 300–1000 m and bring evidence showing that such mats are widespread along the Mediterranean continental margins. Our findings hint at the potential of the FeOB to alter sediment iron bioavailability to the water column. Moreover, we provide indirect evidence for the presence of significant ferrous iron fluxes from the sediment to the sediment-water interface at the marginal areas.

## Materials and Methods

No specific permissions were required for the locations/activities used in this study. We confirm that the field studies did not involve endangered or protected species. The samples were collected from an unprotected area: GPS coordinates defining the study area are: 32°44. 0945′ North, 34°47. 0162′ East; 32°42. 2631′ North, 34°33. 5556′ East; 32°35. 3227′ North, 34°42. 9271′ East; 32°34. 7468′ North, 34°44. 4832′ East.

### ROV imaging and sample collection

**Figure 1:**
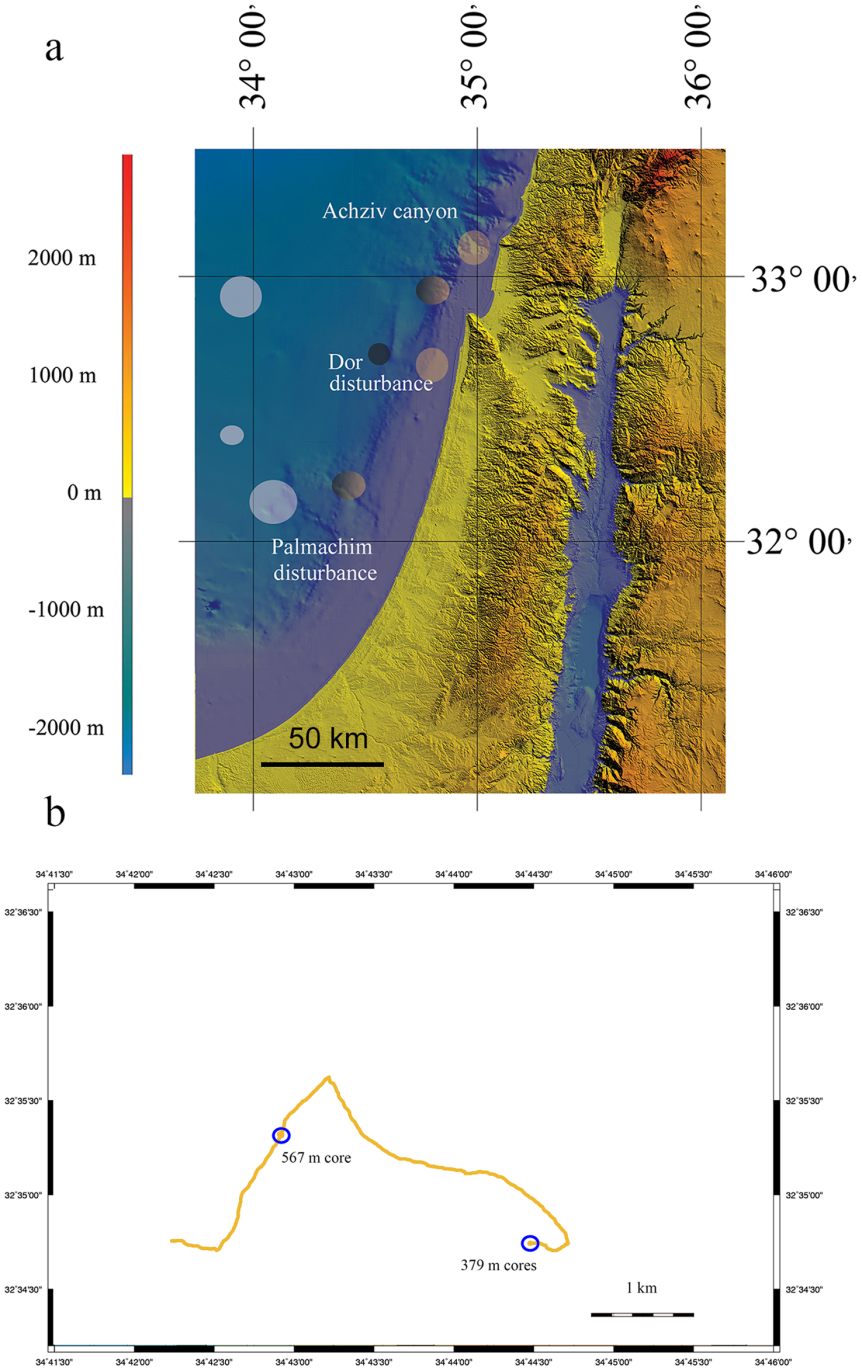
The map of the study sites. a) The bathymetric chart of the Levantine basin displaying areas explored in this study. Yellow circles indicate deposit covered sediments. Yellow to black gradient indicates presence of both orange and black patches. Black circles indicate presence of only black patches. White circles mark areas where no deposits were observed. b) A detailed track of the Dor disturbance survey. The sediment sample locations are shown.

Nautilus E/V is equipped with *Hercules* and *Argus* Remotely Operated Vehicle (ROV) systems, which are able to collect high-resolution video, oceanographic data, and precision sampling. The yellow biofilms at the Levantine sea floor were recorded during two legs of the Nautilus E/V, in September 2010 and November 2011 (NA-009 and NA-019) using the high definition imaging system mounted on the Hercules ROV. The areas explored were the Achziv canyon at the depths of 500–1100 m; two locations in the Acre area, one deep (1200–1700 m) and one shallower (1000–1200 m); Dor disturbance (both legs, 300–900 m) and Palmachim disturbance (both legs, 600–1300 m) [28], [29] (Fig. 1). Each contiguous survey covered approximately 5 km distance (e.g. Fig. 1b), while the field of view diameter was *ca*. 4 m, yielding *ca*. 20000 m^2^ transect area. Bulk sediment (approximately top 5 cm) samples were collected during 2010 *Nautil*us E/V field season from the Dor disturbance at 32°44. 0945′ North, 34°47. 0162′ East at 557 m and from a biofilm at the Achziv Canyon, 32°42. 2631′ North, 34°33. 5556′ East at 1073 m. A subsample from Dor disturbance sediment was frozen for molecular analysis. All other samples were collected during the 2011 *Nautil*us E/V field season. We used 70 mm diameter, 30 cm length cores to sample 9–10 cm profiles at the Dor disturbance location near shore of Israel. A core was taken from the yellow biofilm patch at 32°35. 3227′ North, 34°42. 9271′ East at a depth of 567 m. The biofilm was collected with a pipette and flash-frozen and its pore-water was analyzed for Fe^2+^. Additionally, a biofilm and a control core were collected at 32°34. 7468′ North, 34°44. 4832′ East at a depth of 379 m. These cores were sliced to 1 cm sections, and the sediment was flash-frozen for further processing.

### Microscopy

The flocculent matter from the 567 m core was transferred with a pipette to a slide and observed immediately under various magnifications. The structure of the biofilm was observed with an Imager M2 (Carl Zeiss, Germany) microscope. The images were taken with an AxioCam MRm camera (Carl Zeiss, Germany).

### pH and O_2_ measurements

The pH and O_2_ measurements were performed immediately upon the retrieval of the 567 m core. The intact core with the *in situ* headspace water was subjected to a gentle air bubbling to maintain steady state oxygenation. The FeOB biofilms were easily disturbed during manipulation; hence the profiles were not taken specifically through the biofilm. The pH and O_2_ concentration within the sediment were measured with pH-100 and O_2_-100 microsensors (Unisense, Denmark), respectively, according to manufacturer’s instructions. We utilized a manually operated micromanipulator to obtain the profile at 1 mm resolution. The pH and O_2_ concentrations were recorded at each depth following the stabilization of the signal. The signal was amplified with a Microsensor Multimeter (Unisense, Denmark) and processed in the SensorTrace Basic 3.0 software (Unisense, Denmark). Only one profile was obtained to allow immediate further processing of the core.

### Fe^2+^ and total Fe determination

The pore fluids were extracted by centrifugation under N_2_ atmosphere. Filtered subsamples were assayed immediately by ferrozine reagent [30] and Fe^2+^ in the pore water was determined spectrophotometrically [31]. Total Fe in the biofilm was also determined with the ferrozine assay [31] as follows: 100 µl biofilm subsample was acidified with 1 mol l^−1^ HCl overnight, followed by reduction with hydroxylamine hydrochloride prepared in a solution of analytical grade HCl. Following the ferrozine reagent addition, ammonium acetate solution adjusted to pH 9.5 was added to buffer pH. Only technical replicates were performed, due to limited availability of samples. The sensitivity of this assay is 0.1 nmol l^−1^ [32], hence our results are several orders of magnitude above the detection limit.

### DNA isolation, amplification and sequencing

DNA was isolated from flash-frozen sediments or biofilm, using PowerSoil DNA Isolation Kit (MoBio, Carlsbad, CA, USA) following manufacturer’s instructions. The template DNA was amplified using GoTaq Green Master Mix (Promega, Madison, WI, USA) with addition of 1 µl bovine serum albumin per 50 µl reaction in T100 thermal cycler (Bio-Rad, Hercules, CA, USA). The 16S rRNA gene was amplified using 27F-CM and 1492-R primers [33], yielding ∼1450 bp product; the 23S rRNA gene was amplified with broad specificity forward primer L-0858-a-S-21 and zetaproteobacteria specific L-C-Zeta-1611-A-22 reverse primer [6], yielding ∼750 bp product. The PCR conditions were as follows: denaturation at 94°C for 30 s, annealing at for 30 s 50 °C and extension at 72°C for 1min (1.5 min for 16S) for 35 cycles. PCR products were purified using the Wizard SV Gel and PCR Clean-Up System (Promega, Madison, WI, USA) and subsequently cloned in the pGEM-T Easy vector (Promega, Madison, WI, USA) using *Escherichia coli* JM109 (Promega, Madison, WI, USA) as a host. The sequencing was performed by HyLabs (Israel) using pGEM-T specific primers T7 and SP6.

### Bacterial tag-encoded FLX amplicon pyrosequencing (bTEFAP) and data analysis

16S rRNA gene was amplified using 16S universal Eubacterial primers 104F: 5′ GGC GVA CGG GTG AGT AA; and 530R: 5′ CCG CNG CNG CTG GCA C [34]. A single-step 30 cycle PCR using HotStarTaq Plus Master Mix Kit (Qiagen, Valencia, CA, USA) were used under following conditions: denaturation at 94 °C for 30 s; annealing at 53 °C for 40 s and elongation at 72 °C for 1 min for 28 cycles. Following PCR, all amplicon products from different samples were mixed in equal concentrations and purified using Agencourt Ampure beads (Agencourt Bioscience Corporation, MA, USA). The samples were sequenced utilizing 454 GS FLX titanium (Roche, Penzberg, Germany) following manufacturer’s guidelines. The data derived from the sequencing was processed using a proprietary analysis pipeline [35]–[38] at MR DNA (Shallowater, TX, USA). Barcodes and primers were deleted from the sequences, short sequences < 200bp were removed, sequences with ambiguous base calls were removed, and sequences with homopolymer runs exceeding 6bp were removed. Sequences were denoised and chimeras were removed using custom software [39]. Operational taxonomic units were defined after removal of singleton sequences, clustering at 3% divergence (97% similarity). OTUs were then taxonomically classified using BLASTn against a curated Greengenes database [40] and compiled into each taxonomic level. A total of 1659 valid sequences were established, yielding 97 OTUs.

### Phylogenetic analysis

Evolutionary history was deduced using the maximum likelihood method based on Kimura 2-parameter model [41] (+*I* for 454 16S rRNA genesequences (333 positions), +*G* for 23S rRNA gene sequences (798 positions)) and Data Specific model [42] (+*I*, 1298 positions) for the cloned 16S rRNA gene sequences. The bootstrap consensus tree inferred from 1000 replicates is taken to represent the evolutionary history of the taxa analyzed [43]. Initial trees for the heuristic search were obtained automatically as follows: When the number of common sites was less than 100 or less than one-fourth of the total number of sites, the maximum parsimony method was employed; otherwise BIONJ method with MCL distance matrix was used. Codon positions included were 1st+2nd+3rd+Noncoding. Evolutionary analyses were conducted in MEGA5 [44]. All sequences were submitted to GeneBank under accession numbers KF199322-KF199336, KF651136-KF651143.

## Results

### The distribution of biofilm patches deduced from the ROV imagery

**Figure 2:**
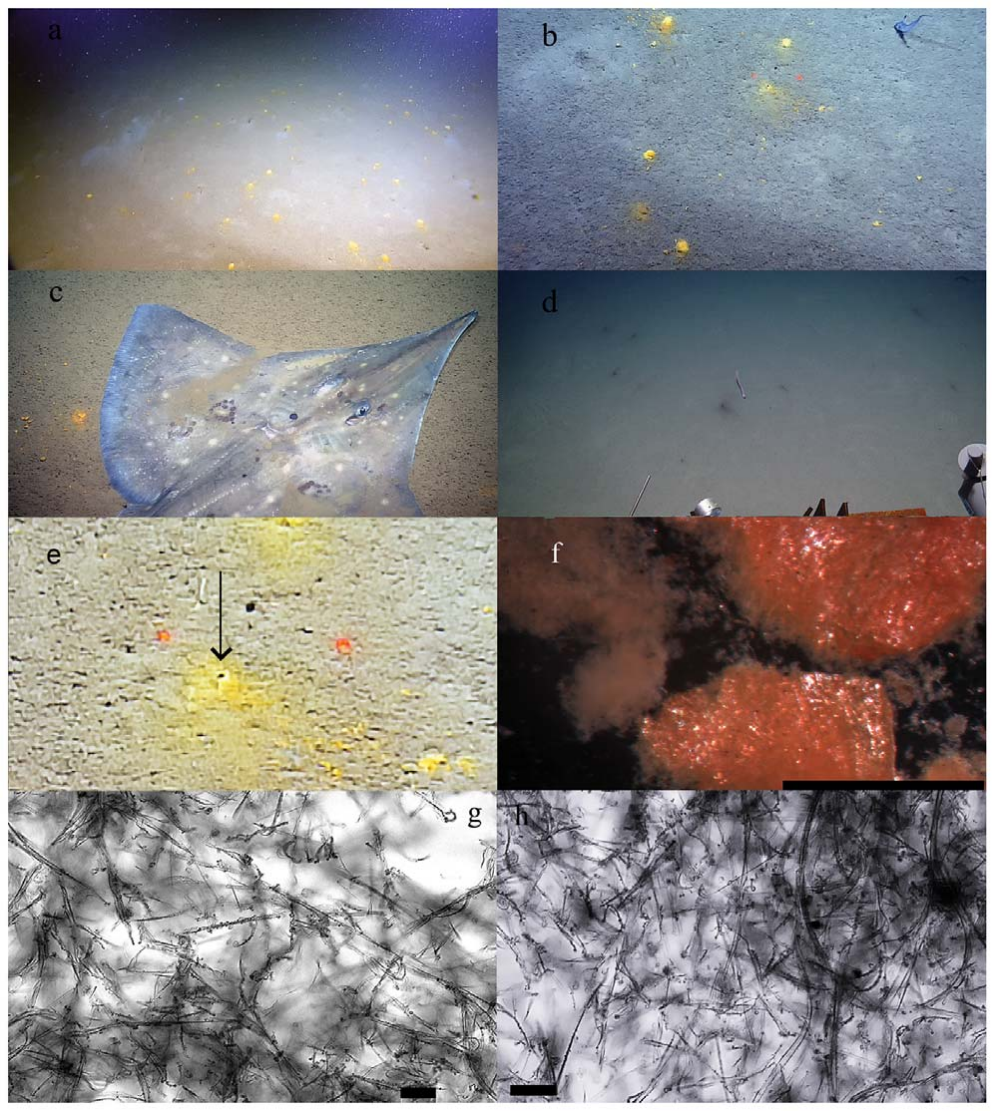
Images of the FeOB patchy mats observed in this study. a) Biofilm patches at the sediment-water interface of Dor disturbance. b) *In situ* close up image of the deposits, the distance between two red dots is 10 cm. c) *Dipturus* sp. re-suspending the deposits. d) Black-colored deposits in at 1000 m depth offshore Israel. e) Close up image of the FeOB patch. The arrow points at potential burrow opening at the core of the patch. f) Microscopic image of the deposits *ex situ* (scale bar is 0.5 mm). g, h) Microscopic images of the deposits (scale bar is 20 µm).

Sediments at 300–800 m areas at the Achziv canyon, Dor disturbance and the Palmachim disturbance (Fig. 1), were covered by sporadically distributed patches of yellow-orange colored flocculent material (Fig. 2a). The size of each biofilm varied from 1 to 5 cm, yet detached particles were scattered over an approximated 10 cm diameter from the biofilm center (Fig. 2b). This phenomenon was consistent throughout the *ca*. 5 km surveys at all explored locations of the latter depth range. The maximum density of the patches was 7 units m^−2^, resulting in approximately 3% coverage of the sediment surface by the biofilm. We noticed that the biofilm was easily disturbed and moved by the ROV’s motion or by biological activity, such as fish movement (Fig. 2c, supplementary video). Moreover, we observed the loss of coloration at depths greater than 800 m at the Achziv canyon, Acre and Palmachim locations (Fig. 2d). Below 800 m the coloration of patches turned black, the biofilm material became less flocculent and no longer easily disturbed (see Fig. S2 for the micrograph of the latter).

### Microscopic observation

At 8x magnification, we observed packed matter with rusty coloration (Fig. 2f). A complex matrix was revealed after a closer inspection at 63x magnification, including transparent organic filaments and darker matrix of higher density (Fig. 2g, h). Small (less than 1 µm) particles were attached along the matrix. Some details from this matrix resembled the stalks with attached iron oxide particles, as found in the Fe-oxidizing zetaproteobacteria [6], [22], [45].

### Physical and chemical parameters of the underlying sediments

**Figure 3:**
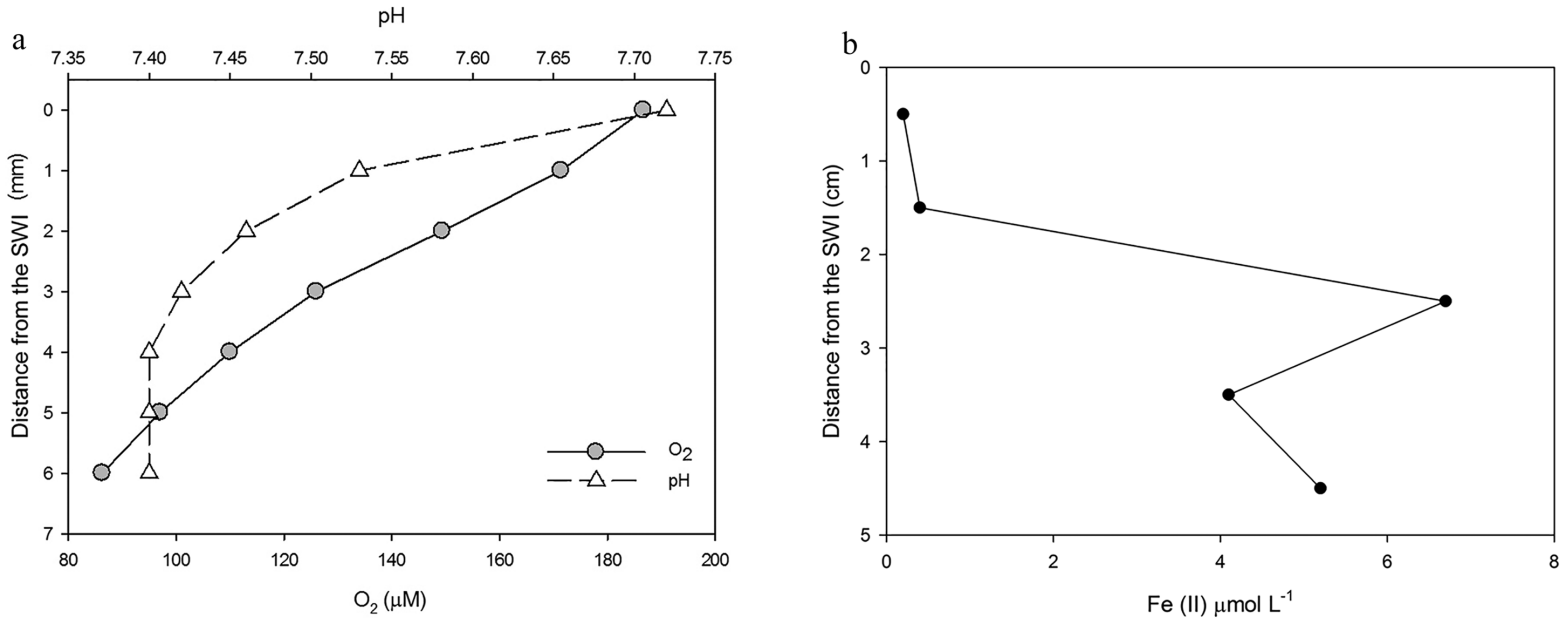
Geochemical parameters of the sampled sediments. a) pH and O_2_ profiles next to the sediment-water interface in the core collected at the depth of 567 m in Dor disturbance. b) Fe^2+^ profile in the same core. (pH and O_2_ were measured at 1 mm resolution, Fe^2+^ was measured at 1 cm resolution.)

The oxygen profile was determined only at the top 6 mm layer in the intact core retrieved from 567 m Dor disturbance sediment. The oxygen dropped from 187 µmol L^−1^ to 83 µmol L^−1^. The pH also decreased throughout the 6 mm profile, from 7.72 to 7.40, stabilized from 4 to 6 mm (Fig. 3a). Fe^2+^ concentration was 0.2–0.4 µmol L^−1^ at first 2 cm of the sediment, followed by a maximum of 6.7 µmol L^−1^ at 2.5 cm below sediment-water interface (Fig. 3b). The total acid leachable iron concentration within the biofilm reached a value of 15.8±0.1 mmol L^−1^.

### Ζetaproteobacteria within the biofilm at the sediment-water interface

In order to establish the phylogenetic relationship of the main biofilm bacteria, *ca.*1450 bp 16S rRNA gene fragments derived from the Sanger sequencing were analyzed. This allowed for more accurate phylogenetic clustering of the studied bacteria than a phylogenetic analysis of shorter (*ca.* 400 bp) sequences derived from bTEFAP [46]. The analysis of the bacterial population from the biofilm revealed the dominance (9 out of 15 sequences) of zetaproteobacterial candidate, related (98% similarity) to the iron oxidizers found in back-arc hydrothermal fields of the Southern Mariana Trough [5] (Fig. S1a). Moreover, it was identical to the zetaproteobacterial candidate sequence retrieved from a FeOB biofilm from the Dor disturbance in 2010. DNA extracted from surface biofilm was also amplified with zetaproteobacteria specific primers for 23S rRNA gene, resulting in determination and identification of this sequence in our sample (Fig. S1b), related to *Mariprofundus* sp. GSB2 (95% similarity) [6].

### Biofilm bacterial population by bTEFAP

**Figure 4:**
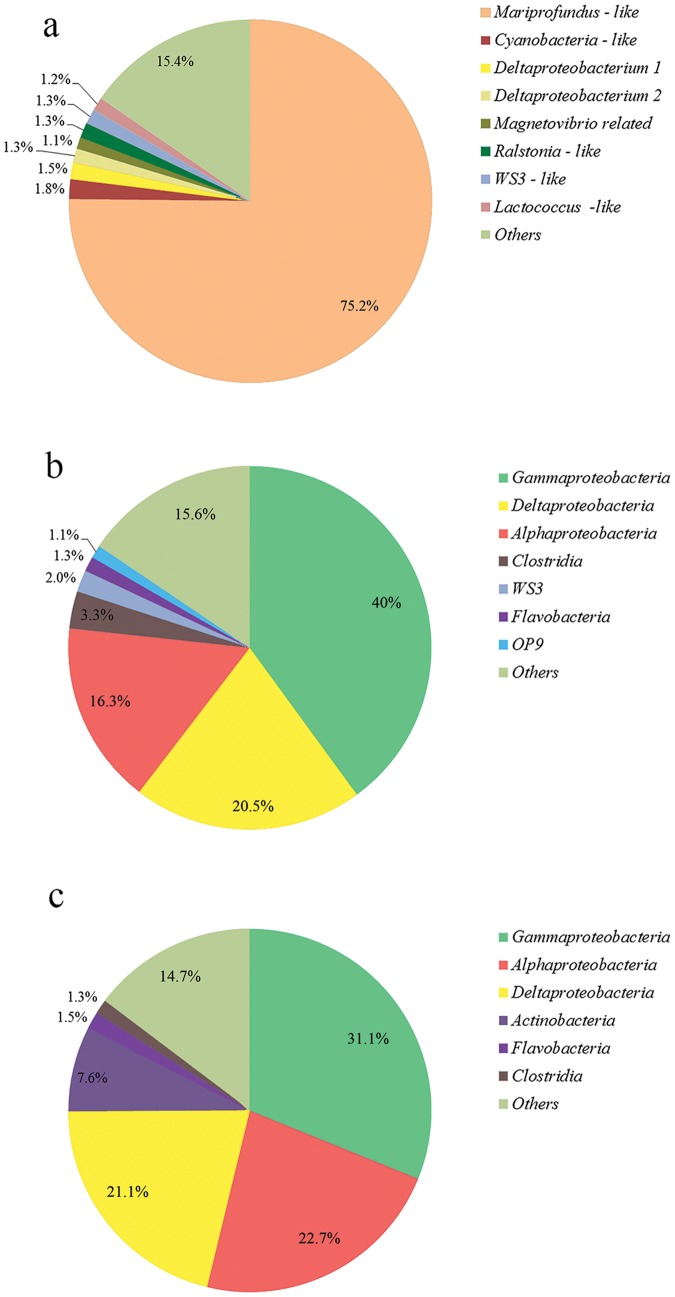
Relative abundance of major bacterial groups derived from bTEFAP analysis. a) Relative abundance of major microbial OTUs within the deposit collected from the 567 m Dor core. b) Relative abundance of major microbial classes within the 1 cm below the sediment-water interface section in the core taken from the deposit at the depth of 379 m in Dor disturbance. c) Relative abundance of major microbial classes within the 1 cm below the sediment-water interface section in the core taken from the non-deposit (control) sediment at the depth of 379 m in Dor disturbance.

**Figure 5:**
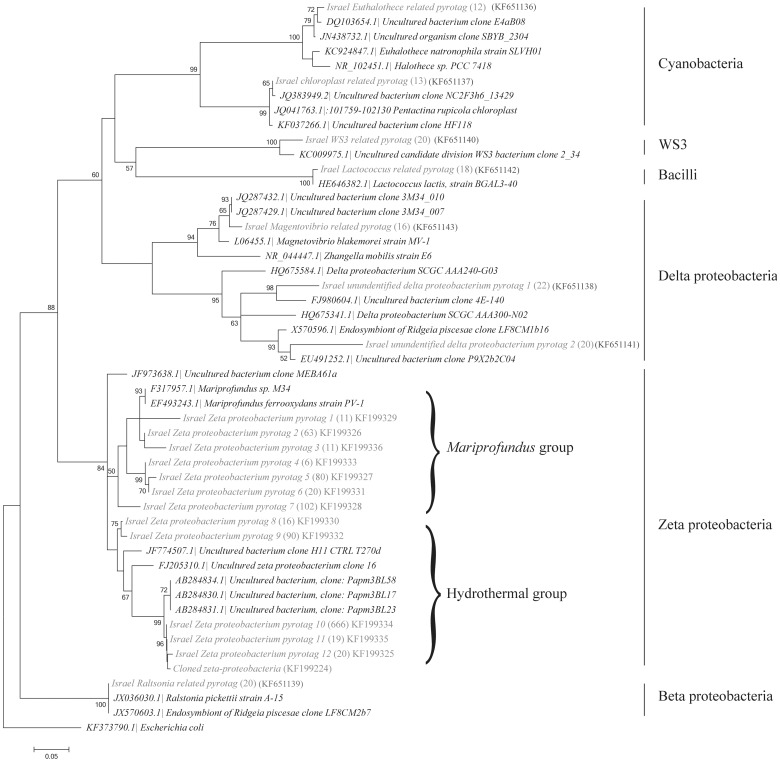
Molecular phylogenetic analysis by maximum likelihood method of most common 16S rRNA pyrotags in the FeOB sample (grey color) and related sequences from the NCBI database. The number of pyrotags yielded for the respective OTU is shown in the brackets. *Escherichia coli* 16S sequence is used as an out-group. The numbers at nodes are bootstrap percent values based on 1000 resamplings. The scale bar corresponds to the number of substitutions per site.

Zetaproteobacterial PYROTAGSs dominated the biofilm (75.2% out of 1659 verified pyrotags) (Fig. 4). Two major zetaproteobacterial groups were derived based on clustering (Fig. 5). One group, including 33% of the zetaproteobacterial pyrotags, clustered with *Mariprofundus* spp., while the second group, including 67% of the zetaproteobacterial pyrotags, clustered with sequences from hydrothermal fields of the Southern Mariana Trough [5]. Other common bacteria, representing more than 1% of total bacterial sequences, included *Ralstonia pickettii* (Betaproteobacteria) – like pyrotags; three deltaproteobacterial pyrotags (one of them was most closely related to *Magnetovibrio blakemorei*); *Lactococcus* – like pyrotags; candidate division WS3 pyrotags and Cyanobacteria – like OTUs. Moreover, we have analyzed the relative abundance of bacterial pyrotags in sediment sections 1 cm below the biofilm and 1 cm sediment-water interface from the control core. No zetaproteobacterial pyrotags were detected in either of these sections, while alpha, delta and gammaproteobacterial pyrotags were dominant, summing up to ∼75% of total pyrotags in the respective samples (Fig. 4b, c).

## Discussion

Our survey of Israel’s continental margin benthos revealed that numerous yellow-orange patches existed within the 300–800 m depth range of the examined areas. These patches were defined by high total iron concentration and by the presence of iron-oxidizing zetaproteobacteria, previously described from hydrothermal vents, exposed oceanic crusts, hydrocarbon seeps and iron deposits. One study determined that zetaproteobacteria were present within brackish sediments [11]. This is the first report describing FeOB populations at deep continental margins lacking hydrothermal or hydrocarbon seepage. Although we were unable to determine conclusively the autotrophic nature of zetaproteobacterial biofilms, we suggest that these biofilms may provide added production to the benthic ecosystem based on the previously determined potential of zetaproteobacteria to fix CO_2_ [1], [2].

The phylogenetic analysis revealed two major clusters of zetaproteobacteria, one related to *Mariprofundus* genus, and a second more abundant cluster related to hydrothermal vent zetaproteobacteria from the Pacific Ocean. The metabolic preferences and factors dictating the relative abundance of the latter groups are still unclear. Other bacterial OTUs had relative abundance less than 2%, although the phylogenetic affiliation of some of them hints at the existence of iron-related microcosm. We have found pyrotags genetically identical to *Ralstonia pickettii*, bacterium previously reported from environments with high metal concentrations [47]. Moreover, pyrotags associated with magnetotactic genus *Magnetovibrio* [48] were found. Although the presence of cyanobacteria related pyrotags within the biofilm is surprising, their source can be the refractory part of marine snow. Moreover, several pyrotags were related to algal chloroplasts. We have previously determined similar sequences from the sediment-water interface at various locations in the deep eastern Mediterranean (data not shown). Hence, they may represent a large array of phytoplankton organisms, especially those protected by a hard cell wall due to an increased chance of preservation in the water column and higher sinking velocities [49].

Based on the hypothesis stating that the bioturbating/bioirrigating organisms’ burrows can induce the ferrous iron transport to the sediment surface [12], we can speculate that such burrows reach the sediment surface below the FeOB biofilm, providing reduced iron to zetaproteobacteria. Tubular hard structures, observed within the inactive black spot bulk sediment sample from 1076 m at the Achziv Canyon (Fig. S2), are potential relict burrows with iron-manganese precipitate walls. This possibly explains the patchy appearance of the biofilm on the seafloor. The Fe^2+^ is most likely produced in 2.5 cm below the sediment-water interface, where its concentration is ∼30 times higher compared to 0.5 cm below the biofilm, and its aided transport is a prerequisite for sufficient flux of Fe^2+^ to sediment surface.

Interestingly, at water depth of 800–1300 m biofilm patches were observed, although their black coloration and lack of flocculence may indicate loss of zetaproteobacterial activity and precipitation of manganese with iron oxides or pyrite formation. Several patches in transitional state (yellow to black) were also present (Fig. S3a, b). No biofilms were observed between 1300–1700 m. The flux of organic matter and benthic infaunal biomass decrease with depth [50]–[52], therefore the increased organic load in shallow sediments can provide a larger pool of electrons for respiration compared to deeper sediments, producing larger quantities of reduced metabolites. This organic load can also support larger populations of bioturbating metazoans that may include sedentary Polychaeta families such as Spionidae, Apharetidae, Pectinariidae and Maldanidae, potentially responsible for the enhancement of Fe^2+^ transport. Hence, a barrier disabling the sediments from providing reduced metabolites to the overlaying water can be produced by insufficient organic matter flux to the seafloor. The seasonal fluctuations dependent on the primary productivity in the photic zone may explain the inactive iron oxidation state of 800–1300 m sediments. Both 2010 and 2011 legs of the *Nautilus* E/V exploration in Levantine basin took place in late summer prior to water column mixing and it should be noted that the depth of biofilm activity would be expected to increase following the spring phytoplankton bloom.

Bioturbation/bioirrigation, sediment resuspension and chemical processes at the sediment-water interface were suggested previously as important mechanisms dictating the fluxes of sediment iron into the water column [53]. In this study we established that the reduced Fe can reach the sediment surface and given the biological resuspension of sediment (Fig 2c, Supp. Video) and deep water mixing, can be transported up the water column. A prerequisite for the ability of iron to travel up the water column is the neutral buoyancy of the iron-containing particle. This can be achieved by detachment of small, colloidal iron particles. Moreover, the association of larger iron particles with positively buoyant organic matter can yield the same result [54], [55]. Based on existing data [56] we estimate that winter sea surface temperatures (SST) below 15°C are sufficient to mix the water column to depths greater than 400 m. The average winter SST in the eastern Mediterranean fluctuates around 15.5 °C, while the lowest average temperature of 15°C was detected during the winter of 1992 [56]. During the last decade, there is an increasing trend of SST values in the eastern Mediterranean Sea [56]. The enhanced SST is expected to decrease iron supply from the sediment surface due to shallower mixing depth [57]. On the contrary, increased temperature of eastern Mediterranean deep water sources can result in the uplift of deep water [58] and increased nutrient flux from the sediment surface to the photic zone. Another positive effect of the global change on the nutrient supply may be the rare, extremely cold winter temperatures that were recently detected in this region [59]. Based on several models, their frequency may increase in the future [60]. In such case, the significance of the sediment-water interface iron as a bioavailable iron source for the water column may increase due to enhanced mixing during an extremely cold event.

**Figure 6:**
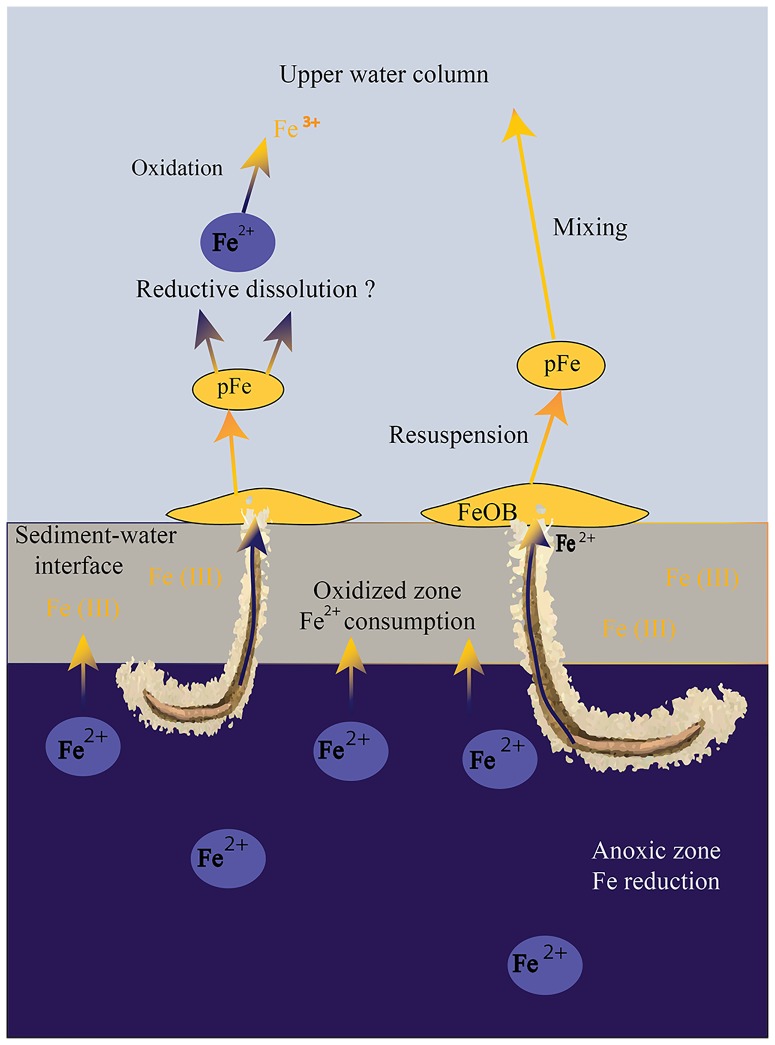
A conceptual model describing formation of the FeOB deposits and potential of the latter to contribute new iron to the water column. Orange to blue color gradient describes reductive process; blue to orange color gradient describes oxidative process. Briefly, Fe (III) oxides are important electron acceptors within the anoxic zone, forming Fe^2+^. Fe^2+^ is diffusively transported to the upper oxygenated sediments, where it is being oxidized back to Fe^3+^. The bioturbating/bioirrigating organisms enhance the transport of Fe^2+^ and allow it to reach the sediment surface, where it is oxidized by zetaproteobacterial FeOB. The flocculent FeOB deposits are easily disturbed and resuspended by larger fauna, being a source of particulate Fe to the water column. Particulate Fe can be reduced inside reducing microenvironments in the water column, becoming bioavailable.

Unfortunately, water column iron profiles are still unavailable in the Levantine basin, and present Mediterranean sampling is limited to shallow (<10 m) or deep (>1500 m) stations [61], [62]. Nonetheless, data from a Weddell Sea station, defined by proximity to the continental shelf and bathymetry resembling our study, suggests iron enrichment close to the sediment-water interface [63], verifying that sediment is a potential source for the water column iron. The bioavailability of the resuspended particulate iron originating from the sediment-water interface can be altered by biologically mediated processes such as microbial reductive dissolution [16], [64]–[67] in the water column (Fig. 6). Iron minerals produced by the stalk forming FeOB are attached to carboxyl-rich polysaccharides [22], providing the organic ligands necessary for the reductive dissolution.

In the report we show that iron-oxidizing bacteria precipitate the ferrous iron at the sediment-water interface along large areas of the continental margin, potentially altering its solubility. The fate of iron precipitated by the zetaproteobacteria is still unclear and its bioavailability may play a crucial role in the future of east Mediterranean ecology under the global change.

## Conclusions

The “plain” sediments at continental margins constitute a heavily understudied area of deep sea research. In this study we report the presence of patchy FeOB mats, abundant in the surveyed locations, unaffected by hydrothermal or hydrocarbon seepage, along the Levantine continental margin. Zetaproteobacterial FeOB, represented by two main phylotypic groups derived from the 16S rRNA gene phylogenetic analysis, constitute the dominant fraction of bacterial population within the biofilms. We suggest that Fe^2+^ concentration sufficient to maintain sediment-water interface FeOB community is most likely achieved by enhancement of pore water upward flux by bioturbating/bioirrigating infauna. Given the potential of FeOB to fix carbon and alter iron chemistry, they may play an important role in the marine environment, affecting both benthic and water column ecosystems.

## Supporting Information

Figure S1 **Molecular phylogenetic analysis by maximum likelihood method of cloned bacterial genetic markers.** a) Evolutionary history of the 16S rRNA small subunit gene. Gamma-proteobacterial 16S sequence is used as an out-group. b) The evolutionary history of 23S rRNA large subunit gene. *Nitrosomonas* sp. 23S rRNA gene sequence is used as an out-group. The numbers are bootstrap percent values based on 1000 resamplings. The scale bar corresponds to the number of substitutions per site.
(TIF)

Figure S2 **Microscopic images of precipitated material within sediments from a black spot.** a) Biogenic tubes. b) Solid particles. Scale bar is 0.5 mm.
(TIF)

Figure S3 **Additional images of FeOB deposits.** a) A deposit in transitional state. b). Deposits next to a metal wreck have both yellow-orange and black colorations. c) A spot in the moddle of FeOB deposit that can be an opening of metazoan burrow. d) Disturbance and resuspention of FeOB deposit by deep-sea *Pleuronectiformes* specimen.
(TIF)

Video S1 **Re-suspension of deposit-covered sediment by** ***Dipturus*** **specimen.**
(MOV)
