# Supplementary figures and images for: First Evidence for the Presence of Iron Oxidizing Zetaproteobacteria at the Levantine Continental Margins
Source: PLoS One. 2014 Mar 10;9(3):e91456. doi: 10.1371/journal.pone.0091456 (PMC3948872; doi:10.1371/journal.pone.0091456)

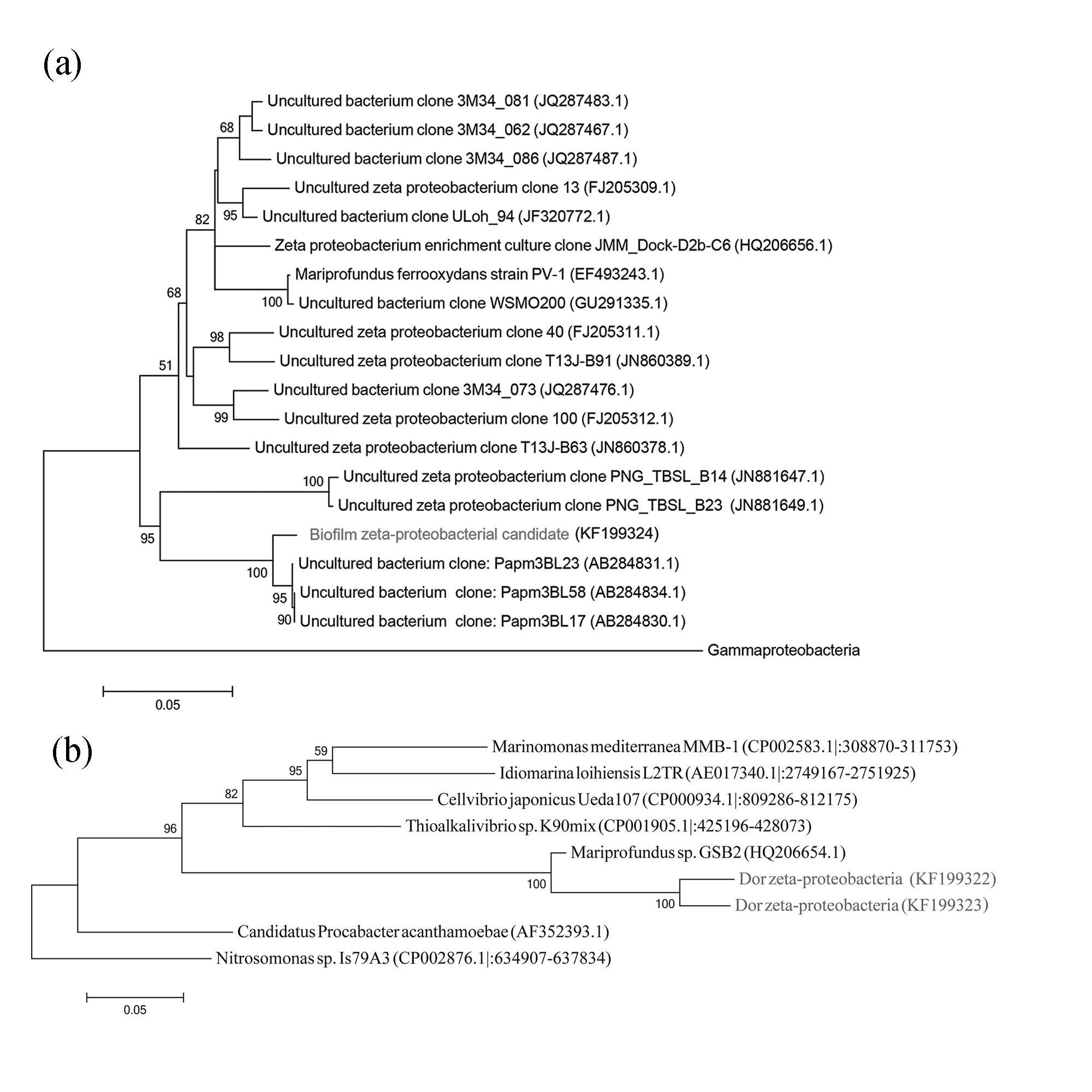

Supplement: Figure S1 — Molecular phylogenetic analysis by maximum likelihood method of cloned bacterial genetic markers. a) Evolutionary history of the 16S rRNA small subunit gene. Gamma-proteobacterial 16S sequence is used as an out-group. b) The evolutionary history of 23S rRNA large subunit gene. Nitrosomonas sp. 23S rRNA gene sequence is used as an out-group. The numbers are bootstrap percent values based on 1000 resamplings. The scale bar corresponds to the number of substitutions per site. (TIF) [file pone.0091456.s001.tif]

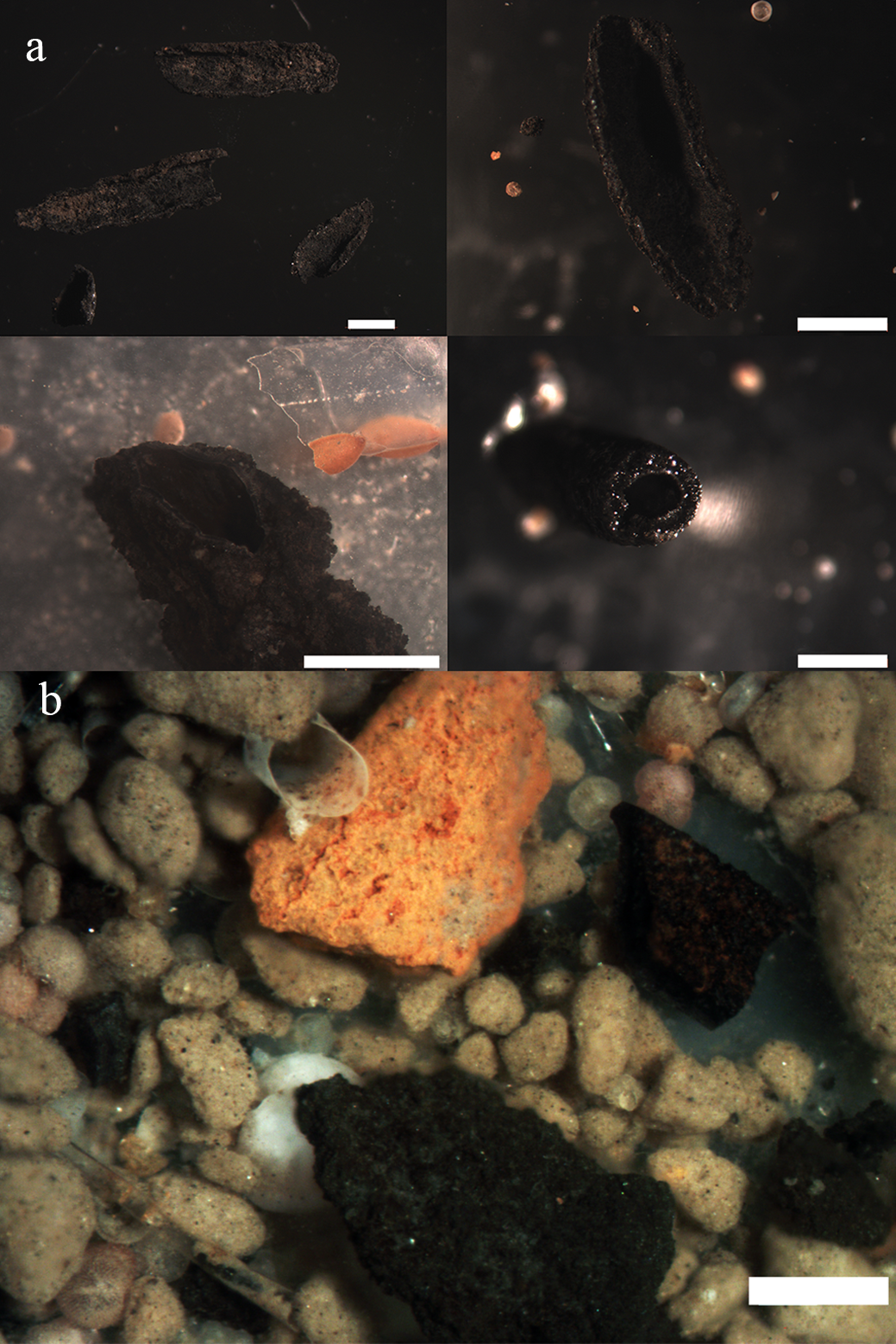

Supplement: Figure S2 — Microscopic images of precipitated material within sediments from a black spot. a) Biogenic tubes. b) Solid particles. Scale bar is 0.5 mm. (TIF) [file pone.0091456.s002.tif]

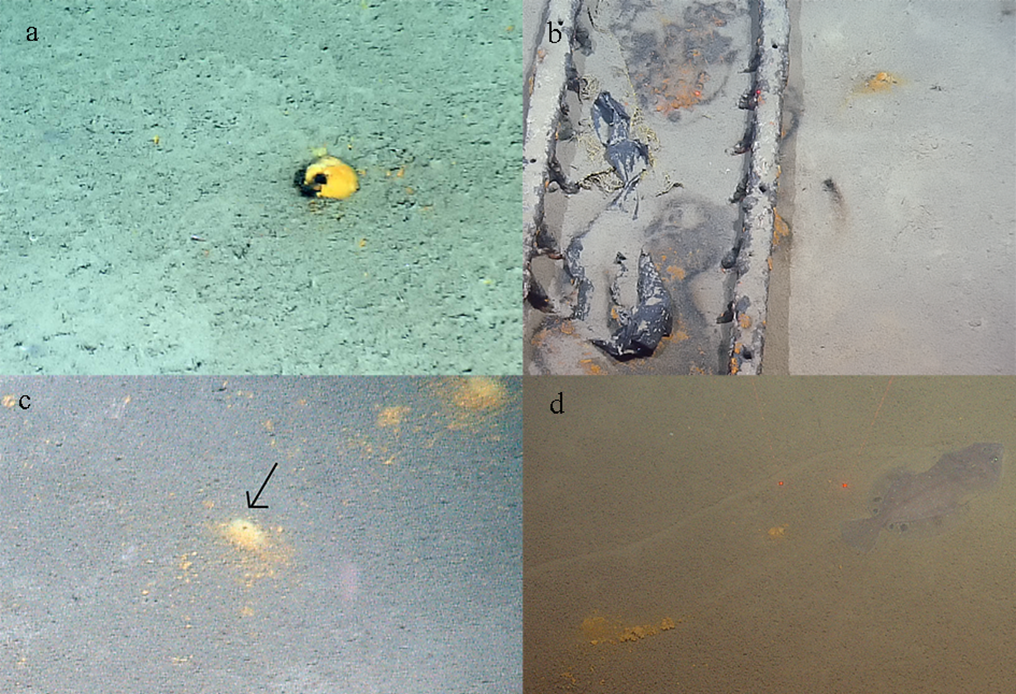

Supplement: Figure S3 — Additional images of FeOB deposits. a) A deposit in transitional state. b). Deposits next to a metal wreck have both yellow-orange and black colorations. c) A spot in the moddle of FeOB deposit that can be an opening of metazoan burrow. d) Disturbance and resuspention of FeOB deposit by deep-sea Pleuronectiformes specimen. (TIF) [file pone.0091456.s003.tif]
